# Background Intestinal ^18^F-FDG Uptake Is Related to Serum Lipid Profile and Obesity in Breast Cancer Patients

**DOI:** 10.1371/journal.pone.0141473

**Published:** 2015-11-02

**Authors:** Hai-Jeon Yoon, Han-Na Kim, Yeojun Yun, Yemi Kim, Ae-Na Ha, Hyung-Lae Kim, Bom Sahn Kim

**Affiliations:** 1 Department of Nuclear Medicine, Ewha Womans University, School of Medicine, Seoul, Republic of Korea; 2 Department of Biochemistry, Ewha Womans University, School of Medicine, Seoul, Republic of Korea; 3 Medical Research Institute, Ewha Womans University, School of Medicine, Seoul, Republic of Korea; University Hospital Essen, GERMANY

## Abstract

**Background:**

This study investigated the relationships between background intestinal uptake on ^18^F–FDG PET and cardio-metabolic risk (CMR) factors.

**Methods:**

A total of 326 female patients that underwent ^18^F–FDG PET to determine the initial stage of breast cancer were enrolled. None of the patients had history of diabetes or hypertension. The background intestinal uptake on PET was visually graded (low vs. high uptake group) and quantitatively measured using the maximal standardized uptake value (SUV_max_). SUV_max_ of 7 bowel segments (duodenum, jejunum, ileum, cecum, hepatic flexure, splenic flexure, and descending colon-sigmoid junction) were averaged for the total bowel (TB SUV_max_). Age, body mass index (BMI), fasting blood glucose level (BST), triglyceride (TG), cholesterol, high density lipoprotein (HDL), and low density lipoprotein (LDL) were the considered CMR factors. The relationships between background intestinal ^18^F–FDG uptake on PET and diverse CMR factors were analyzed.

**Results:**

The visual grades based on background intestinal ^18^F–FDG uptake classified 100 (30.7%) patients into the low uptake group, while 226 (69.3%) were classified into the high uptake group. Among CMR factors, age (*p* = 0.004), BMI (*p*<0.001), and TG (*p*<0.001) were significantly different according to visual grade of background intestinal ^18^F–FDG uptake. Quantitative TB SUV_max_ showed significant positive correlation with age (*r* = 0.203, *p*<0.001), BMI (*r* = 0.373, *p*<0.001), TG (*r* = 0.338, *p*<0.001), cholesterol (*r* = 0.148, *p* = 0.008), and LDL (*r* = 0.143, *p* = 0.024) and significant negative correlation with HDL (*r* = -0.147, *p* = 0.022). Multivariate analysis indicated that BMI and TG were independent factors in both visually graded background intestinal ^18^F–FDG uptake (*p* = 0.027 and *p* = 0.023, respectively) and quantitatively measured TB SUV_max_ (*p* = 0.006 and *p* = 0.004, respectively).

**Conclusion:**

Increased background intestinal ^18^F–FDG uptake on PET may suggest alteration of lipid metabolism and risk of cardio-metabolic disease in non-diabetic and non-hypertensive breast cancer patients.

## Introduction

Positron emission tomography (PET) using ^18^F-fluorodeoxyglucose (FDG) is a widely used functional imaging modality for glucose metabolism. This modality was initially developed for tumor imaging because tumors have a higher ^18^F-FDG intake than normal cells. However, applications for PET have expanded, and this technique is now being used in non-tumor pathophysiology. Accordingly, there is increased interest in the variable range of physiologic ^18^F-FDG uptake in normal subjects.

The intestine, which is involved in diverse metabolic pathways, also presents a variable range of physiologic ^18^F-FDG uptake. However, physiologic ^18^F-FDG uptake along the intestinal tract is not fully understood, and several hypotheses have suggested roles of smooth muscle cells, superficial mucosal cells, lymphoid tissue, and ^18^F-FDG excretion in the stool [1–4].

Based on our clinical experience, we have frequently identified high and diffuse background intestinal ^18^F-FDG uptake in obese patients. Obesity is a multifactorial metabolic disorder that increases the likelihood of other cardio-metabolic disease. Therefore, we conducted a retrospective study to elucidate the relationships of background intestinal ^18^F-FDG uptake with diverse cardio-metabolic risk (CMR) factors.

## Materials and Methods

### Subjects

We retrospectively reviewed the FDG PET/CT database at Ewha Womans University Cancer Center for Women and selected a breast cancer cohort in order to establish a sufficient number of subjects. This study was approved by the institutional review board (IRB) of the Ewha Womans University Mokdong Hospital. All procedures performed in studies involving human participants were in accordance with the ethical standards of the IRB and with the 1964 Helsinki declaration and its later amendments or comparable ethical standards. Waiver of consent was obtained from the IRB for all patients and all of the data was anonymized prior to analysis. Our database yielded 425 breast cancer women who underwent ^18^F–FDG PET/CT for initial staging work-up between July 2012 and March 2015. There were no patients who underwent neoadjuvant chemotherapy before PET/CT. Because the purpose of this study was to investigate the relationships of background intestinal ^18^F–FDG uptake with CMR factors in non-CM disease, we excluded 99 patients with diabetic mellitus (DM) or hypertension (HTN). A total of 326 non-diabetic, non-hypertensive patients were included in the final statistical analysis. None of the 326 patients were currently suffering or had recently suffered from any inflammatory disease, such as inflammatory bowel disease, infectious colitis, cholangitis, rheumatism, etc., or had taken any medication due to such a condition at the time of PET/CT.

### Data Collection

Social history of smoking or alcohol use was determined by medical chart review. The anthropometric data of patients, including height and weight, were available from the PET center patient record, which was completed at the time of visit for the PET study. Body mass index (BMI) was calculated using height and weight.

The standard laboratory data before initial treatment were reviewed, and levels of triglyceride (TG), cholesterol, high density lipoprotein (HDL), and low density lipoprotein (LDL) were determined from the lipid profile.

### Assessment of background intestinal ^18^F–FDG uptake

For ^18^F–FDG PET/CT, patients were instructed to fast for at least six hours before intravenous administration of 5.18 MBq/kg FDG and then to rest for one hour before the scan. Blood glucose level was measured before FDG administration and confirmed to be <140 mg/dL. A CT scan without contrast agent was obtained first, and then a PET scan was obtained from the base of the skull to the thigh, using a Siemens Biograph mCT with 128 slice CT (Siemens Medical Solutions, Erlangen, Germany). Low-dose CT was used as a transmission map for attenuation correction and was performed with a CARE Dose system (Siemens Medical Solutions, Erlangen, Germany). The spatial resolution at the center of the PET was 2.0 mm full width at half maximum (FWHM) in the transaxial direction and 2.0 mm FWHM in the axial direction. The acquisition parameters for PET images were 3D emission scan and 2 min scan/bed positionx5-7 positions. PET images were reconstructed to 200x200 matrices and 3.4 mmx3.4 mm pixel size with 3.0 mm slice thickness using a 3D OSEM iterative algorithm (2 iterations and 21 subsets) with time of flight (TOF) and point spread function (PSF).

Qualitative and quantitative assessment of background intestinal ^18^F–FDG uptake was performed by two readers. Analysis was carried out in consensus, and the interpreters were strictly blinded to CMR data. For qualitative assessment, the range of background intestinal ^18^F–FDG uptake was dichotomized as low and high. Patients with lower background intestinal ^18^F–FDG uptake of the whole intestine compared to that of the liver were classified as the low uptake group, while patients with background intestinal ^18^F–FDG uptake with at least one segment equal to or greater than that of the liver were classified as the high uptake group. For quantitative assessment, the maximum and mean standardized uptake value (SUV_max_ and SUV_mean_) in different bowel segments were measured after placement of a three-dimensional volume of interest (VOI) [3, 4]. SUV_mean_ was measured with a margin threshold of 40% SUV_max_. Background intestinal ^18^F–FDG uptake of the small bowel was measured at the third duodenum, jejunum, and distal ileum loop. Background intestinal ^18^F–FDG uptake of the large bowel was measured at the cecum, hepatic flexure, splenic flexure, and descending colon-sigmoid junction. Then, SUVs of different bowel segments were averaged for a measure of the total bowel (TB SUV_max_ and TB SUV_mean_).

### Statistics

Data are presented as the mean ± SD with a 95% confidence interval (CI). *P* values less than 0.05 were regarded as significant. All statistical analyses were performed using SPSS software version 18.0 (SPSS Inc., Chicago, IL, USA). The relationships between anthropometric data (height, weight, and BMI), demographic data (age), and laboratory data (BST and lipid profile) with background intestinal ^18^F–FDG uptake were evaluated using the independent t-test for dichotomized groups, while Pearson’s correlation analysis was used for continuous SUV. The interpreting criteria for the magnitude of correlation (*r*) were: <0.1, very weak; 0.1–0.3, weak; 0.3–0.5, moderate; 0.5–1.0, strong. Regression analysis was used to evaluate the association between each CMR factor and background intestinal ^18^F–FDG uptake. Then, multivariate regression analysis was performed to determine the independent effect of each variable on background intestinal ^18^F–FDG uptake.

## Results

Data are provided as mean ± SD with 95% confidence interval (CI). None of the 326 patients had a history of social smoking or alcohol use. Mean patient age was 47.2 ±9.2 years (range 28–79 years), and mean height, weight, and calculated BMI were 158.3±5.4 cm (range 144.0–173.0), 57.6±8.2 kg (range 35–88), and 23.0±3.2 (range 15.1–34.8), respectively. The average time interval between lipid profile and PET scan was 1.5±9.2 (range 0–32) days. The BST level was 99.0±10.9 mg/dl (range 75.0–188.0), and the TG and cholesterol levels were 96.9±54.8 mg/dl (range 10.0–393.0) and 191.5±36.6 mg/dl (range 87.0–345.0), respectively. The HDL and LDL levels were 53.2±22.6 mg/dl (range 26.0–340.0) and 113.4±36.5 mg/dl (range 37.0–237.0), respectively.

### Qualitative assessment

Based on visual grade of background intestinal ^18^F–FDG uptake, 100 (30.7%) patients were included in the low uptake group, and 226 (69.3%) were included in the high uptake group. Among the CMR factors, age (*p* = 0.004), BMI (*p*<0.001), and TG (*p*<0.001) were significantly different by visual grade of background intestinal ^18^F–FDG uptake. The detailed data are presented in Table 1.

**Table 1:** Cardio-metabolic risk factors according to the visual grade of background intestinal ^18^F–FDG uptake.

	Low uptake	High uptake	*p* value
Demographic data			
Age (years)	45.2±7.4	48.1±9.8	0.004*
Anthropometric data			
Height (cm)	158.6±5.5	156.9±6.1	0.012*
Weight (kg)	56.1±6.9	58.5±9.0	0.012*
Body mass index	22.0±2.6	23.5±3.4	<0.001*
Laboratory data			
BST (mg/dl)	100.3±13.5	98.4±9.6	0.149
Triglyceride (mg/dl)	80.5±44.6	104.2±57.5	<0.001*
Cholesterol (mg/dl)	186.0±34.4	193.9±37.4	0.075
HDL (mg/dl)	54.9±14.0	52.4±25.8	0.403
LDL (mg/dl)	109.0±40.0	115.6±34.7	0.189

BST, blood sugar testing; HDL, high-density lipoprotein; LDL, low-density lipoprotein

*p<0.05

**Fig 1:**
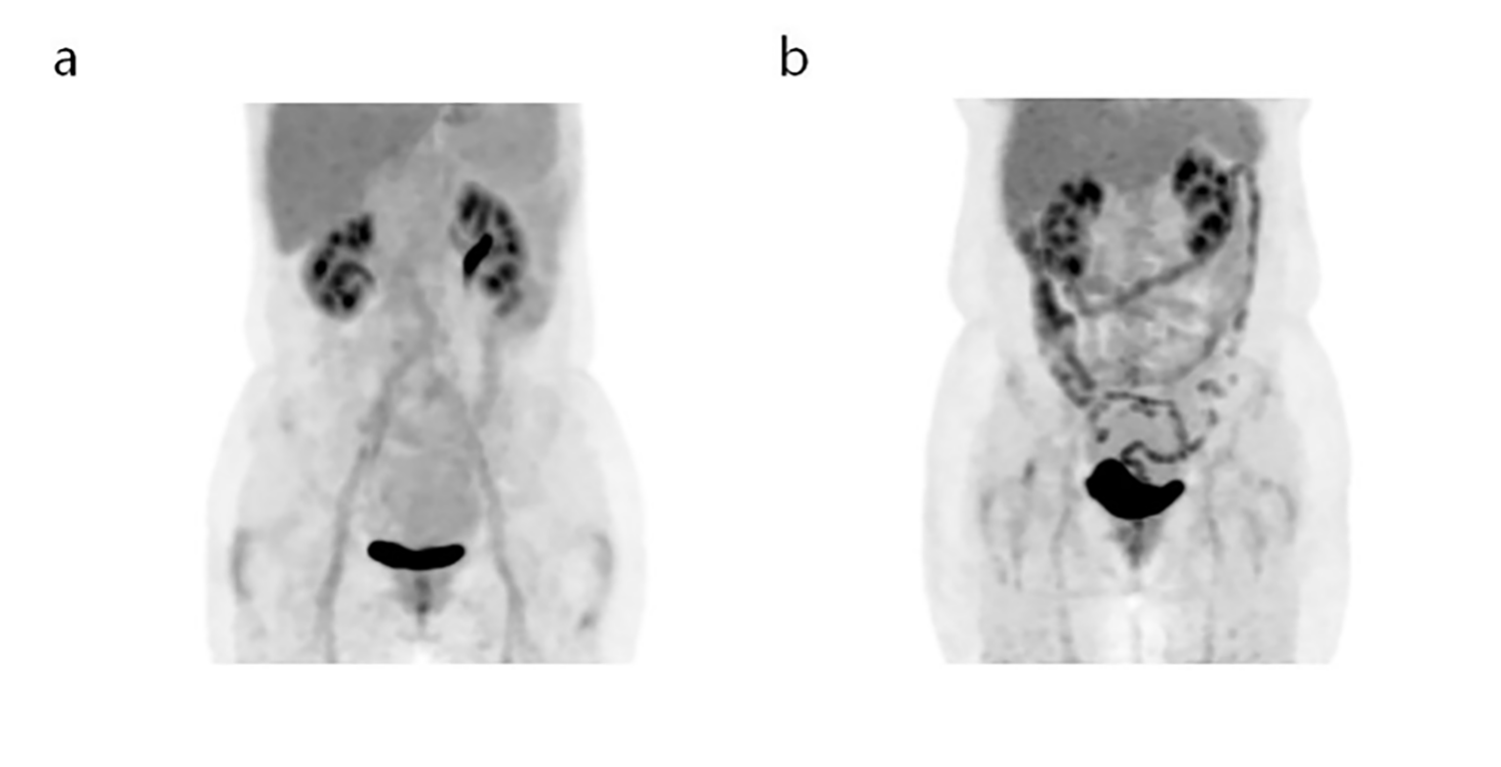
(a) A 57-year-old women with right breast cancer underwent ^18^F–FDG PET/CT. Mild ^18^F–FDG uptake (inferior to the liver) resulted in her being classified into the low uptake group. Her BMI was 20.0, and triglyceride level was 45 mg/dL. (b) A 64-year-old women with left breast cancer underwent ^18^F–FDG PET/CT. Intense ^18^F–FDG uptake along the intestine was classified into the high uptake group. Her BMI was 27.3, and triglyceride level was 393 mg/dL.

On univariate regression analysis, older age (*p* = 0.009), higher BMI (*p*<0.001), and higher TG (*p* = 0.001) were significant factors for increased background intestinal ^18^F–FDG uptake on PET. The cholesterol level showed equivocal significance (*p* = 0.077). The detailed data are presented in Table 2. When the multiple regression analysis was performed regarding the three significant variables and one equivocal variable on univariate analysis, BMI and TG were independent factors for background intestinal ^18^F–FDG uptake on PET (*p* = 0.027 and *p* = 0.023, respectively). Representative cases for the differences in CMR factors according to visual grade of background intestinal ^18^F–FDG uptake on PET are shown in Fig 1.

**Table 2:** Results of univariate and multivariate logistic regression analysis.

	Coefficient	Odds ratio (95% CI)	*p* value
**Univariate**			
Age	0.037	1.037 (1.009–1.066)	0.009*
Body mass index	0.150	1.161 (1.068–1.263)	<0.001*
BST	-0.015	0.985 (0.964–1.006)	0.156
Triglyceride	0.010	1.010 (1.004–1.016)	0.001*
Cholesterol	0.006	1.006 (0.999–1.013)	0.077
HDL	-0.005	0.995 (0.984–1.007)	0.425
LDL	0.005	1.005 (0.997–1.013)	0.189
**Multivariate**			
Age	0.014	1.014 (0.983–1.045)	0.386
Body mass index	0.102	1.108 (1.011–1.213)	0.027*
Triglyceride	0.007	1.007 (1.001–1.013)	0.023*
Cholesterol	0.002	1.002 (0.994–1.009)	0.644

HDL, high-density lipoprotein; LDL, low-density lipoprotein

*p<0.05

### Quantitative assessment

**Fig 2:**
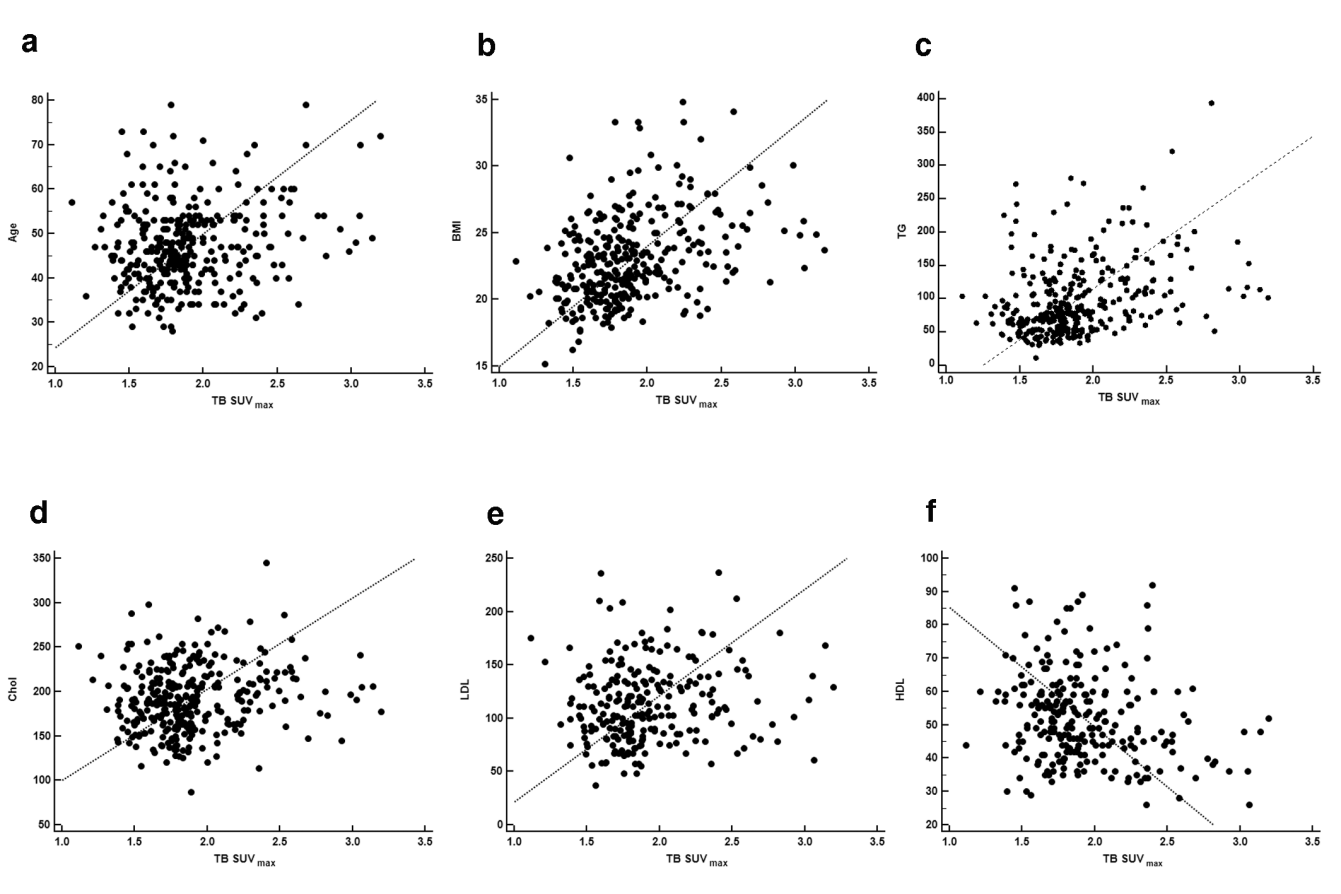
Scatter plots of age (a), body mass index (b), triglyceride (c), cholesterol (d), low-density lipoprotein (e), and high-density lipoprotein (f) according TB SUV_max_.

The overall TB SUV_max_ was 2.0±0.5 (range 1.2–4.0) and TB SUV_mean_ was 1.7±0.4 (range 1.0–3.1). According to Pearson’s correlation analysis, TB SUV_max_ showed significant positive correlation with age (*r* = 0.203 and *p* <0.001, Fig 2A), BMI (*r* = 0.373 and *p*<0.001, Fig 2B), TG (*r* = 0.338 and *p*<0.001, Fig 2C), cholesterol (*r* = 0.148 and *p* = 0.008, Fig 2D), and LDL (*r* = 0.143 and *p* = 0.024, Fig 2E) and significant negative correlation with HDL (*r* = -0.147 and *p* = 0.022, Fig 2F). TB SUV_mean_ also showed significant positive correlation with age (*r* = 0.175 and *p* = 0.001), BMI (*r* = 0.333 and *p*<0.001), TG (*r* = 0.321 and *p*<0.001), cholesterol (*r* = 0.151 and *p* = 0.007), and LDL (*r* = 0.136 and *p* = 0.033) and significant negative correlation with HDL (*r* = -0.131 and *p* = 0.040).

On univariate regression analysis, older age (*p*<0.001), higher BMI (*p*<0.001), higher TG (*p*<0.001), higher cholesterol (*p* = 0.008), lower HDL (*p* = 0.022), and higher LDL (*p* = 0.024) were significant factors of increased TB SUV_max_ on PET. Regarding TB SUV_mean_, age (*p* = 0.001), BMI (*p*<0.001), TG (*p*<0.001), cholesterol (*p* = 0.007), HDL (*p* = 0.040), and LDL (*p* = 0.033) were also significant factors. When multiple regression analysis was performed with the six significant variables on univariate analysis, BMI and TG were independent factors of TB SUV_max_ (*p* = 0.006 and *p* = 0.004, respectively) and TB SUV_mean_ (*p* = 0.007 and *p* = 0.017, respectively) on PET. The detailed data are presented in Table 3.

**Table 3:** Results of univariate and multivariate regression analyses.

	Coefficient	95% CI	*p* value
**TB SUV**_**max**_			
**Univariate**			
Age	0.010	0.005–0.016	<0.001*
Body mass index	0.053	0.039–0.068	<0.001*
BST	0.00001	-0.005–0.005	0.996
Triglyceride	0.003	0.002–0.004	<0.001*
Cholesterol	0.002	0.001–0.003	0.008*
HDL	-0.003	-0.006–0.000	0.022*
LDL	0.002	0.000–0.003	0.024*
**Multivariate**			
Age	0.004	-0.003–0.010	0.323
Body mass index	0.028	0.008–0.048	0.006*
Triglyceride	0.002	0.001–0.003	0.004*
Cholesterol	-0.00002	-0.002–0.002	0.981
HDL	-0.002	-0.005–0.001	0.137
LDL	0.001	-0.001–0.003	0.249
**TB SUV**_**mean**_			
**Univariate**			
Age	0.007	0.003–0.012	0.001*
Body mass index	0.038	0.027–0.050	<0.001*
BST	-0.001	-0.004–0.003	0.712
Triglyceride	0.002	0.001–0.003	<0.001*
Cholesterol	0.002	0.000–0.003	0.007*
HDL	-0.002	-0.004–0.000	0.040*
LDL	0.001	0.000–0.003	0.033*
**Multivariate**			
Age	0.002	-0.004–0.007	0.596
Body mass index	0.023	0.006–0.039	0.007*
Triglyceride	0.001	0.000–0.002	0.017*
Cholesterol	0.000	-0.001–0.002	0.693
HDL	-0.001	-0.004–0.001	0.175
LDL	0.001	-0.001–0.002	0.332

HDL, high-density lipoprotein; LDL, low-density lipoprotein

*p<0.05

## Discussion

Cardio-metabolic risk (CMR) is defined as a cluster of risk factors that increase the risk of cardiovascular disease and diabetes [5, 6]. The CMR factors encompass traditional risk factors, such as age, sex, hypertension, insulin resistance, dyslipidemia, and smoking, as well as emerging risk factors, such as abdominal obesity as measured by waist circumference. This study considered age, fasting glucose, and lipid profile (TG, cholesterol, LDL, and HDL) as CMR factors. We also considered BMI as an indicator of obesity. Our results demonstrated that background intestinal ^18^F–FDG uptake on PET showed significant positive correlations with age, BMI, and levels of TG, cholesterol, and LDL and a significant negative correlation with level of HDL. On multivariate analysis, BMI and TG level were independent factors of background intestinal ^18^F–FDG uptake.

The first investigation about factors that influence intestinal ^18^F-FDG uptake was reported by Yasuda et al. in 1998 [7]. They reported that sex, age, and bowel condition affected intestinal ^18^F-FDG uptake. However, they could not find any relationship between free fatty acid (FFA) level and intestinal ^18^F-FDG uptake. Their result is somewhat discordant with our study, which indicated a strong relationship between lipid profile and intestinal ^18^F-FDG uptake. The difference in study populations (e.g., age and sex) and metabolites (e.g., TG and FFA) may contribute to the disagreement.

Other study groups have reported that the oral hypoglycemic agent metformin increased intestinal ^18^F-FDG uptake [3, 4]. The hypoglycemic effect of metformin enhances glucose utility of the intestinal enterocyte and seems to affect intestinal ^18^F-FDG uptake in diabetic patients [8–10]. However, variable ranges of background intestinal ^18^F-FDG uptake are frequently observed in non-diabetic patients as well.

Recently, the possible role of intestinal bacteria on background intestinal ^18^F-FDG uptake has been suggested. Franquet et al. demonstrated that pretreatment with rifaximin, which is an antibiotic agent retained in the intestinal lumen, significantly reduces background intestinal ^18^F-FDG uptake [11]. It has been reported that rifaximin alters intestinal bacterial population [12]. Considering the action of rifaximin on intestine, the reductions in background intestinal ^18^F-FDG uptake imply the contribution of intestinal bacteria in the mechanism.

Considering the possible contribution of intestinal bacteria to background intestinal ^18^F-FDG uptake, the alteration of gut flora and subsequent modification of host metabolism may induce the interpersonal differences in background intestinal ^18^F-FDG uptake. The gut flora is composed of hundreds of prevalent bacterial species that metabolize xenobiotic compounds, amino acids, and carbohydrates [13, 14]. Increasing evidence indicates that gut microbiota are an environmental factor in host metabolism and innate immunity [15]. Alteration of gut flora contributes to obesity by metabolizing indigestible fiber to absorbable short-chain fatty acids [16–20]. Moreover, bacterial components cause an innate inflammatory response and subsequent disturbance in host metabolism that are related to the pathophysiology of metabolic syndrome, obesity, cardiovascular disease, and diabetes [21, 22].

Based on our objective observation of an increasing trend of background intestinal ^18^F-FDG uptake in obese patients, we concluded relationships between intestinal ^18^F-FDG uptake and diverse CMR factors. According to our results, aged women with obesity and dyslipidemia tend to show high background intestinal ^18^F-FDG uptake on PET. The role of gut flora in the modulation of lipid metabolism may contribute to the strong relationships between background intestinal ^18^F–FDG uptake and obesity and lipid profile. Additional intestinal absorption of short-chain fatty acids metabolized by intestinal bacteria can increase the level of TG and cause obesity. However, intestinal bacteria have not yet been found to directly affect intestinal ^18^F-FDG uptake. Analysis of fecal or mucosal microbiota using 16S ribosomal DNA gene sequencing correlated with intestinal ^18^F-FDG uptake on PET is a promising future study.

This current study indicates the value of background intestinal ^18^F–FDG uptake as a potential surrogate biomarker of CMR. Increased background intestinal ^18^F–FDG uptake on PET suggests not only alteration of lipid metabolism, but also high risk of metabolic syndrome, cardiovascular disease, and diabetes in non-diabetic and non-hypertensive populations. To the best of our knowledge, this is the first report on the relationships of intestinal ^18^F–FDG uptake with CMR factors. Furthermore, this study illuminates a previously unidentified field of research on the clinical significance of intestinal ^18^F–FDG uptake as an imaging biomarker, targeting the gut flora and lipid metabolism of human subjects. Currently, ^18^F–FDG PET is widely used for cancer evaluation, but background intestinal ^18^F–FDG uptake is an obstacle to be overcome. Based on the current investigation, we recommend the evaluation of CMR in a patient with intense bowel uptake who underwent ^18^F–FDG PET for cancer evaluation. However, ^18^F–FDG PET as a CMR screen is not recommended due to the radiation hazard and high costs.

Our study had several limitations. First, the retrospective design is a major limitation. While the known effect of metformin on increased bowel uptake was completely eliminated by excluding diabetic patients, confounding effects caused by other medications were not considered. Despite our careful medical chart review, there is a possibility of missing information about recent or current medications due to minor infections or presence of chronic systemic inflammations at the time of PET/CT. Second, the population was confined to a breast cancer cohort. It is known that obesity is associated with both the risk of breast cancer and the clinical behavior of the established disease [23]. A previous study indicated that intestinal bacteria contribute to the risk of breast cancer through the modulation of estrogen metabolism [24]. Thus, the strong relationships between ^18^F–FDG bowel uptake and obesity and lipid metabolism in this study may be specific to the breast cancer cohort. For generalized application of the results, further study with non-cancerous healthy cohorts should be performed. Third, the population was confined to non-diabetic, non-hypertensive, non-smoking female subjects. Thus, the contribution of sex, hypertension, diabetes, and smoking on ^18^F–FDG bowel uptake could not be investigated. Forth, we considered BMI as an indicator of obesity, even though the standard is central obesity measured by waist circumference. However, due to the retrospective nature of this study, data on waist circumference was not available.

## Conclusions

This study shows that cardio-metabolic risk factors affect background intestinal ^18^F–FDG uptake on PET. Obesity and dyslipidemia were important factors for high background intestinal ^18^F–FDG uptake in non-diabetic and non-hypertensive breast cancer patients. Our findings identify the value of background intestinal ^18^F–FDG uptake as a potential surrogate biomarker of cardio-metabolic risk.
